# Positive Salt Tolerance Modulation via Vermicompost Regulation of SOS1 Gene Expression and Antioxidant Homeostasis in *Vicia* *faba* Plant

**DOI:** 10.3390/plants10112477

**Published:** 2021-11-16

**Authors:** Rehab El-Dakak, Weam El-Aggan, Ghadah Badr, Amira Helaly, Amel Tammam

**Affiliations:** 1 Department of Botany and Microbiology, Faculty of Science, Alexandria University, Alexandria 21511, Egypt; weamelaggan3@gmail.com (W.E.-A.); amel_tammam@yahoo.com (A.T.); 2 Department of Biological Science, Faculty of Science, Elmergib University, Al Khums P.O. Box 40414, Libya; ghadasaad748@gmail.com; 3 Department of Vegetable Crops, Faculty of Agriculture, Alexandria University, Alexandria 21545, Egypt; amira.helaly@alexu.edu.eg

**Keywords:** *Vicia* *faba*, broad bean, salinity, SOS1, stress, vermicompost

## Abstract

Strategic implementation of vermicompost as safe biofertilizer besides defensing saline soils offer dual function solving problems in developing countries. The current study aims to utilize vermicompost (VC) for amelioration of 200mM NaCl in *Vicia faba* Aspani cultivar and investigate the molecular role of salt overly sensitive pathway (SOS1). The experiment was conducted following a completely randomized design with three replicates. Treatments include 0; 2.5; 5; 10; 15% dried VC intermingled with soil mixture (clay: sand; 1:2) and/or 200 mM NaCl. The results show that salinity stress decreased broad bean fresh and dry weight; and K^+^/Na^+^. However, malonedialdehyde and H_2_O_2_ contents; increased. Application of 10% VC and salinity stress increases Ca^2+^ (41% and 50%), K^+^/Na^+^ (125% and 89%), Mg^2+^ (25% and 36%), N (8% and 11%), indole acetic acid (70% and 152%) and proteins (9% and 13%) for root and shoot, respectively, in comparison to salt treated pots. Moreover, all examined enzymatic antioxidants and their substrates increased, except glutathione reductase. A parallel decrease in abscisic acid (75% and 29%) and proline (59% and 58%) was also recorded for roots and leaves, respectively. Interestingly, the highly significant increase in gene expression of SOS1 (45-fold) could drive defense machinery of broad bean to counteract 200 mM NaCl.

## 1. Introduction

The unprecedented growth of wastes around the globe is a normal consequence of the escalating population and the quest of rapid economic advance [1]. In developing countries, serious environmental and human health threats have emerged following the decay of biodegradable wastes, producing various noxious gases and leachate [2].

Vermicomposting is a promising eco-friendly technology for the bioconversion of different waste types, which serve as reservoirs of environmental pollution [3]. Nutrient-rich organic compost produced from solid waste by specific earthworms (as *Eisenia foetida* sp.) which can improve plant health and fertility. Therefore, issues such as casting out safely organic wastes, crop production enhancement and sustainability of food security can be overcome by the green technique of vermicomposting [4].

*Vici faba* are a popular legume, consumed worldwide as an important protein source for human and animal nutrition [5]. In Egypt, broad bean is one of the most important winter pulses, being cultivated from the north to the deep south, although it attains the least self-sufficiency values compared to wheat, maize, and barley [6]. The aerial parts of *V. faba* are rich in protein, supplying humans with most of the essential elements necessary for life [7]. Additionally, soil physical traits, accompanied by microbial activity, are improved via the capacity of *V. faba* to facilitate the solubilization of phosphorous to neighboring crops [8]. Moreover, the nitrogen-fixing capacity of *Vicia faba* as a leguminous plant has raised its value during crop rotation [9].

Soil salinization is an agriculturally critical subject and has endangered targets of sustainable development linked to nutrition, food security, and resource conservation. Physio-chemical characteristics of soil, in addition to plant metabolism, are subjected to the deleterious effect of the increasing levels of salinity-stress [10]. Salinity problem significantly affects broad bean productivity, lipids content, proteins, nucleic acids and inhibits enzyme activities of carbon and nitrogen metabolism [11]. Additionally, salinity has a negative influence on the abundance and distribution of soil-dwelling microorganisms. Nowadays, conventional approaches do not fit well with the research trials to control the salinity issue [10]. Traditional management techniques of agroecosystems mainly rely on adding nutrients and water to fill the gap of nutrient deficiencies, but not to remove the accumulated salts. This exacerbates soil problems caused by fertilizer-induced salinization [12]. Therefore, modern salinity management techniques such as vermicomposting are mostly promoted [10]. It is pertinent that organic amendments are able to ameliorate salt affected soils by improving their physical, chemical, and biological conditions for beneficent soil health, boosting crop production [13]. Thus, the aim of the current work was to determine (1) whether the vermicompost application can improve the negative effect of salt stress in Aspani cultivar of *Vicia faba* (2) and if so, which vermicompost combination ratio to soil is required to elicit such salt stress, and (3) finally, investigate the role of vermicompost in controlling SOS1 gene expression, which may guide sodium homeostasis and growth traits to enhance salt tolerance.

## 2. Results

### 2.1. Vermicompost Improves Morphological Traits in Broad Bean Plant

A preliminary survey conducted on the *Vicia faba* plant revealed that 200 mM NaCl is the concentration that causes about 50% growth inhibition, with respect to control. In a parallel survey on vermicompost, five different VC levels were chosen: 0, 2.5, 5, 10, and 15%. To assess the role of vermicompost on morphometric traits, plant height, root, shoot fresh, and dry weights were measured. Under normal conditions, vermicompost significantly increased plant height by (27, 25, 14 and 5% at 2.5, 5, 10 and 15% VC, respectively), root and shoot fresh weight by (52 and 25 at 2.5% VC, respectively), root and shoot dry weight by (36 and 16% at 2.5% VC, respectively) with respect to control.

Salt tolerance indices measured for the morphological traits of broad bean plants revealed that 200 mM NaCl treatment caused a decrease in plant height STI (67%) and shoot fresh weight STI (68%) (Table 1). Results recorded a significant increase in STI of all morphological traits with vermicompost treatments in salt-stressed broad bean plant (2.5 and S, 5 and S in addition to 10% VC and S) with the highest increase at 10% VC salt-stressed plants, whereas 15% VC and S showed a decreasing trend. The percentages of increase with respect to salt-treated plants for 10% VC and S are 64.3, 121.4 and 178.6 for plant height, root and shoot dry weight, respectively.

In another way to evaluate salt tolerance, membership function value (MFV) was calculated (Table 2). Mean MFV is a multiple indicator where the bigger MFV mean, the higher salt tolerance. Results of the current work clarified that after the ranking of mean MFV, broad bean plants fell into three salinity levels: relatively salt sensitive (RSS) at S; 2.5% VC and S (14.479 and 12.020, respectively), relatively salt tolerant (RST) at 5% VC and S; 15% VC and S (51.680 and 60.582, respectively), and relatively highly salt tolerant (RHST) at 10% VC and S, which achieved mean MFV = 91.582. It is interesting to notice that the results of the morphological parameters at 2.5 and 5% VC was found to be very close, whereas almost all traits above 10% VC showed aa decreasing trend.

### 2.2. Vermicompost Maintains Element Homeostasis in Salt-Stressed Broad Bean Plant

Elemental status in the present study involved the investigation of Mg^2 +^, K^+^/Na^+^, and Ca^2+^ contents in broad bean plants (Table 3). Results showed that in NaCl-treated pots, Mg^2+^ content decreases significantly with respect to the non-stress pots (27 and 39% in roots and leaves, respectively). Addition of vermicompost to NaCl-treated pots improves the negative effect on Mg^2+^ content in roots and leaves, especially at 10% VC and S relative to 200 mM NaCl-treatment (25% and 36%, respectively).

The ratio of K^+^/Na^+^ offers insight into ion homeostasis within the plant. Thus, upon exposure to saline treatment, K^+^/Na^+^ ratio decreased significantly in roots and leaves by (70 and 48%, respectively). On the other hand, exposure of broad bean to 10% VC and 200 mM NaCl strengthened K^+^/Na^+^ homeostasis through elevating K^+^ by (95 and 78% for roots and leaves, respectively), while reducing Na^+^ by (12 and 6% for roots and leaves, respectively) compared to salt-stressed plants. This renders K^+^/Na^+^ ratio to increase by 125 and 89% in roots and leaves, respectively.

Calcium response is different from that of Mg^2+^ and K^+^/Na^+^ ratio. Exposure of broad bean plants to salinity stress causes an unexpected increase in Ca^2+^ content in roots and leaves (16 and 32%, respectively). Application of VC to salt-stressed pots caused the highest significant increase of Ca^2+^ content at 10% VC and S by (41 and 50% in roots and leaves, respectively) as compared to salt-treated pots.

### 2.3. Vermicompost Modulates Hormonal Status, Proline, Nitrogen and Protein Content in Broad Bean Plant

To achieve the mechanisms related to osmotic adjustment, The contents of proline, nitrogen, protein, indole acetic acid (IAA) and abscisic acid (ABA) contents were measured at normal and stressed conditions. The results presented in Table 4 show the decline in IAA content of roots and leaves under 200 mM NaCl treatment, which is amounted to 38 and 46%, respectively, compared to control. However, an obvious significant increase in ABA and proline contents is recorded by about 14 and 5-fold for roots, respectively. The corresponding values for leaves were 4 and 6-fold. Nitrogen and protein contents attain a significant decrease expressed by 21 and 18% for nitrogen; 21 and 22% for protein in roots and shoots, respectively.

The positive effect of VC on NaCl-treated broad bean plants appears clearly at 10% VC in the maximum increase in IAA (1.7-fold and 2.5-fold for roots and leaves, respectively), nitrogen, and protein contents (1.08-fold and 1.11-fold; 1.08-fold and 1.13-fold for roots and shoots, respectively), and in the significant decrease in ABA and proline contents when compared with salt-treated plants (0.25-fold and 0.39-fold; 0.41-fold and 0.42-fold, for roots and leaves, respectively).

### 2.4. Vermicompost Reduces Lipid Peroxidation and H_2_O_2_ through the Improvement of Antioxidant Enzyme Activities in Broad Bean Plant

**Figure 1:**
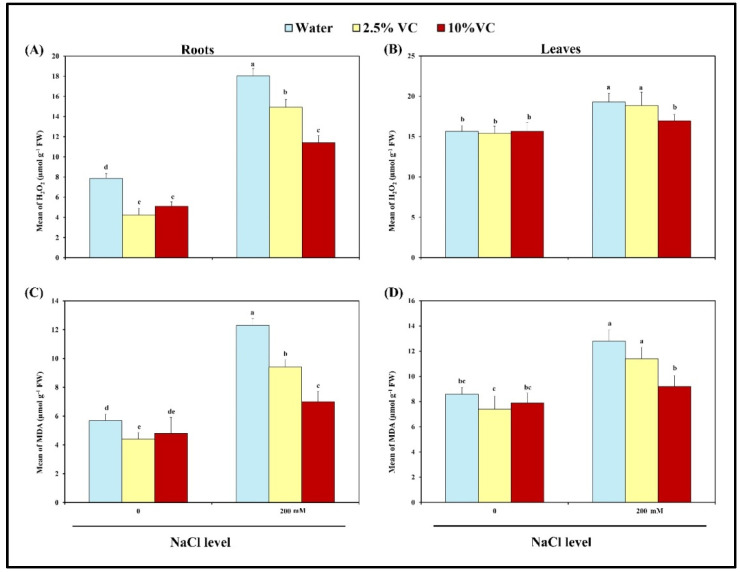
Effects of vermicompost with 2.5 and 10 percentages on hydrogen peroxide (**A** and **B**) and malondialdehyde (**C** and **D**) in the roots and leaves of broad bean plants exposed to 0 and 200 mM NaCl after 30 days of cultivation. Bars represent means and standard deviation of three independent replications (*n* = 3). Different alphabetical letters indicate significant differences among the treatments at *p* < 0.05, according to a Duncan’s multiple range test.

The role of vermicompost against oxidative stress was assessed by determining the level of hydrogen peroxide (H_2_O_2_) and lipid peroxidation in terms of malondialdehyde (MDA) content in addition to the activities of ROS-detoxifying enzymes in roots and leaves of broad bean plants. The results in Figure 1 reveal that H_2_O_2_ and MDA contents increase in response to salt stress in broad bean plants by 128 and 23% in roots, respectively, as compared to control. The corresponding values for leaves were 116 and 49%. This was reversed by all the VC applied to stressed plants, with minimum H_2_O _2_ and MDA values recorded for roots and leaves at 10% VC, compared to salt-treated plants (36.7 and 12.1; 43.1 and 28.1, respectively). Interestingly, vermicompost showed positive effects especially at 2.5% VC in the reduction of H_2_O_2_ and MDA content in roots and leaves (46 and 1.5; 23 and 14%, respectively), even under normal conditions when compared with control.

**Figure 2:**
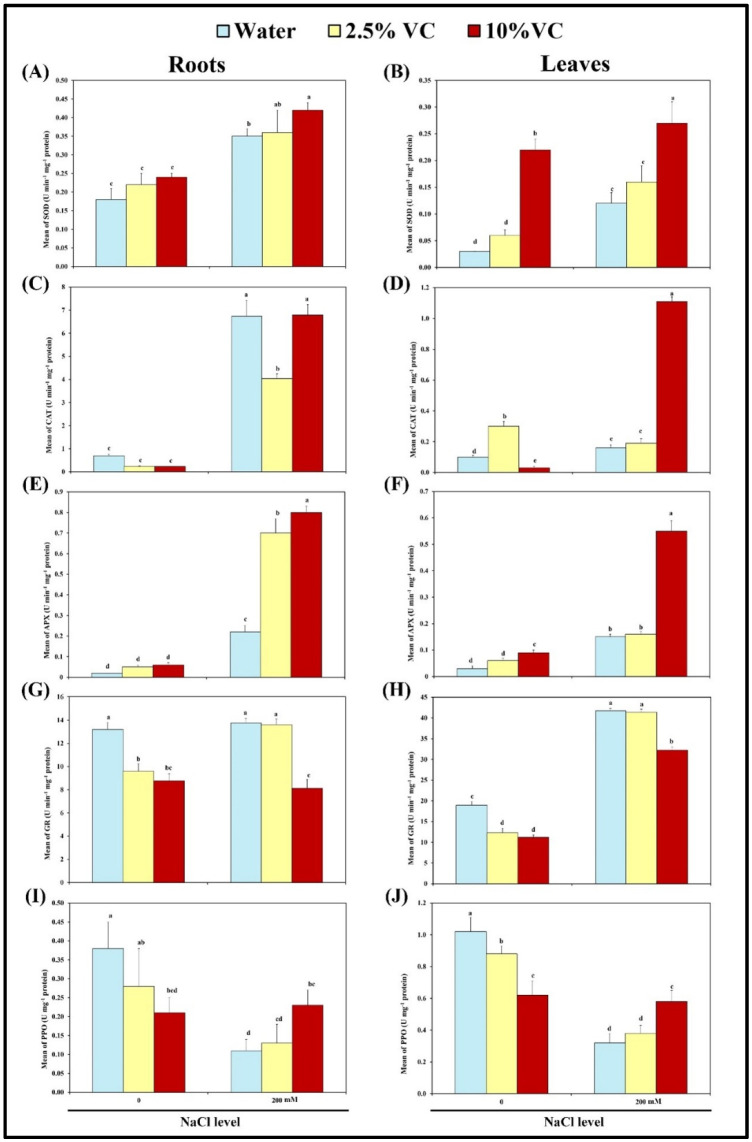
Effects of vermicompost with 2.5 and 10 percentages on superoxide dismutase-SOD (**A**,**B**), catalase-CAT (**C**,**D**), ascorbate peroxidase-APX (**E**,**F**), glutathione reductase-GR (**G**,**H**) and polyphenol oxidase-PPO (**I**,**J**) in the roots and leaves of broad bean plants exposed to 0 and 200 mM NaCl after 30 days of cultivation. Bars represent means and standard deviation of three independent replications (*n* = 3). Different alphabetical letters indicate significant differences among the treatments at *p* < 0.05, according to a Duncan’s multiple range test.

In the current study, broad bean plants accomplished fighting the exerted salinity oxidative stress via enzymatic and non-enzymatic machinery. Antioxidant enzymes superoxide dismutase (SOD), catalase (CAT), ascorbate peroxidase (APX) and glutathione reductase (GR) showed differential responses in broad bean plants treated with salt stress and/or vermicompost (Figure 2A–H). Most of the studied enzymatic antioxidant activities tested in roots and leaves show higher expression under salinity stress; the SOD, CAT and APX activities increase in roots by 2-, 10- and 11-fold, respectively, as compared to the control. The corresponding values for leaves are 4-, 2- and 5- fold, respectively (Figure 2A–F). On the other hand, glutathione reductase shows a 2-fold increase in leaves with respect to the control. Applying 200 mM NaCl to 2.5% and 10% VC pots showed changes in activities of SOD, CAT, APX and GR in roots and leaves compared to salt-treated pots. The results declare that all enzymatic antioxidants exhibited their maximum activities at 10% VC except for GR. The increase in SOD, CAT and APX activities in roots were estimated by about 1.2-, 1- and 4-fold, respectively; the corresponding values for leaves were 2-, 7- and 4-fold, respectively compared with salt-treated plants. Analogously, glutathione reductase activity decreases in roots and leaves by about 60% and 80% of their corresponding salt-treated plant, respectively (Figure 2G,H).

The activity of polyphenol oxidase (PPO) is illustrated in Figure 2I,J. Salt stress treatments resulted in a decrease in PPO activity in roots and leaves by about 71% and 69% of the control value, respectively. In contrast, PPO activity in 200 mM NaCl and VC-treated broad bean plants is significantly increased in all treatments of VC examined, as compared to salt-stressed plants, peculiarly at 10% VC treatment which induced its maximal level in roots (0.30 Umg^−1^ protein) by about a 2.7-fold increase in the salt-stressed plant (Figure 2A–F). The same trend was recorded in leaves as PPO activity was induced significantly to its maximal level (0.83 Umg^−1^ protein) by about a 2.6-fold increase in salt stress.

### 2.5. Vermicompost Modulate the Non-Enzymatic Antioxidantsin Broad Bean Plant

The non-enzymatic antioxidants defense to various abiotic stresses is regulated by the overexpression of the AsA-GSH pathway and phenolic compounds. The contents of ascorbate (AA), dehydroascorbate (DHA), the ratio of AA/DHA, reduced glutathione (GSH), oxidized glutathione (GSSG), the ratio of GSH/GSSG estimated in roots and leaves of *Vicia faba* plant are presented in Table 5. Supplementing growing media with 200 mM NaCl resulted in a significant increase in all the individual values of ascorbate and glutathione content, except for their ratios which are decreased as compared with control. Applying VC to the salt-stress pots caused the highest increase in AA/DHA ratio of broad bean plants at 10% VC and S in roots and leaves by about 10 and 22%, respectively, as compared to salt-treated plants. Analogously, applying 10% VC to stress pots caused a significant increase in GSH/GSSG ratio, amounting to 11 and 40%, respectively.

In salt-treated broad bean plants, phenolic content decreased in roots and leaves by 79 and 75%, respectively, as compared to the control. The additive effects of vermicompost and salt stress together are observed in the noticeable increase in the production of phenolic compounds in comparison with the salt-stressed broad bean plants. The highest increase in phenolic content in the roots and leaves was achieved at 10% VC and amounted to about 100 and 115%, respectively, when compared with the corresponding values for salt- stress plants. The above results reveal that 10% vermicompost has a positive effect on broad bean growth performance under salt stress and were linked to (1) improving growth, N, protein, and IAA contents (2) restriction of Na^+^ uptake for maintaining K^+^/Na^+^ homeostasis, (3) decreased MDA and H_2_O_2_ contents, and (4) activation of an antioxidant system for efficient ROS scavenging, thereby suggesting that VC acts as an effective candidate for designing specific eco- friendly fertilizer to enhance salt stress tolerance of major crops such as broad bean.

### 2.6. Hierarchical Clustering and PCA Analysis Show Positive Interactions between Treatments and Variables

**Figure 3:**
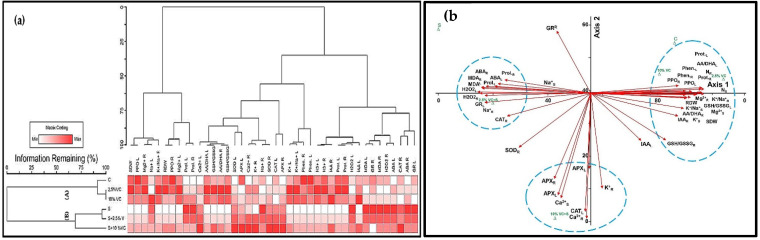
Hierarchical clustering and principal component analysis (PCA) to understand treatment-variable relationships in broad bean plants under both normal and saline conditions. (**a**) The mean values of different parameters were normalized and clustered. Two distinct clusters at the treatment levels (A, B) were detected. Color scale shows the intensity of the normalized mean values of different parameters. (**b**) The whole dataset was analyzed using PCA. The lines originating from the central point of biplots show negative or positive correlations of different variables, where their closeness reveals correlation strength with a particular treatment. The variables included RDW (root dry weight), SDW (shoot dry weight), Ca^2+^ R (calcium root), Ca^2+^ L (calcium leaf), Mg^2+^ R (magnesium root), Mg^2+^ L (magnesium leaf), K^+^ R (potassium root), K^+^ L (potassium leaf), N^3+^ R (nitrogen root), N^3+^ L (nitrogen leaf), Na^+^ R (sodium root), Na^+^ L (sodium leaf), K^+^/Na^+^ R (potassium/sodium root), K^+^/Na^+^ L (potassium/sodium leaf), Prot. R (protein root), Prot. L (protein leaf), MDA R (malondialdehyde root), MDA L (malondialdehyde leaf), H_2_O_2_ R (hydrogen peroxide root), H_2_O_2_ L (hydrogen peroxide leaf), SOD R (superoxide dismutase root), SOD L (superoxide dismutase leaf), CAT R (catalase root), CAT L (catalase leaf), APX R (ascorbate peroxidase root), APX L (ascorbate peroxidase leaf), GR R (glutathione reductase root), GR L (glutathione reductase leaf), AA/DHA R (ascorbic acid/dehydroascorbic acid root), AA/DHA L (ascorbic acid/dehydroascorbic acid leaf), GSH/GSSG R (reduced/oxidised glutathione root), GSH/GSSG L (reduced/oxidised glutathione leaf), Phen. R (phenolics root), Phen. L (phenolics leaf), PPO R (polyphenol oxidase root), PPO L (polyphenol oxidase leaf), IAA R (indoleacetic acid root), IAA L (indoleacetic acid leaf), ABA R (abscisic acid root), ABA L (abscisic acid leaf), Prol. R (proline root), Prol. L (proline leaf).

Average mean values of morpho-physiological and biochemical data obtained from broad bean plants under normal and salinity conditions are used to perform heat map (HM) and hierarchical clustering (HC), then PCA. Hierarchical clustering applied on the treatments reveals their classification into two main groups (Group A and Group B) (Figure 3a). Group A includes control, 2.5 and 10% VC, whereas group B includes treatments: S, 2.5% VC and S in addition to 10% VC and S. It is noted that the applied heat map reflects the trend of the parameters (decreasing or increasing) relative to the matrix code (white or red, respectively).

A principle component analysis (PCA) was performed to find the association between the measured parameters and the different groups of applied treatments (Figure 3b). The two components of PCA (PC1 = 70.86 and PC2 = 17.83) collectively explained 88.70% of data variability. Results reveal the appearance of three groups of association. The first group concerned 2.5 and 10% VC. The second involves 2.5% VC and S. Unexpectedly, 10% VC and 200 mM NaCl achieved a peculiar group of parameters association with relatively the highest trend in comparison to the salt-stressed treatment (namely, Ca^+2^; APX in roots and leaves; together with roots K^+^ and leaves SOD; CAT). Here, such associated parameters are related to attributes such as growth, enzymatic antioxidant, and potassium replacement of sodium ions by effluxion through the common Na^+^/K^+^ ion pump in collaboration with Ca^+2^ role in the SOS1 signaling pathway via the plasma membrane.

The study of HM, HC and PCA revealed that 10% VC and S achieved an independent association group with a number of physiological parameters with the relatively highest values. Down to the last step, molecular validation of the obtained results concerning 10%VC and S was inevitable.

### 2.7. Vermicompost Upregulates Gene Expression of Salt Overly Sensitive (SOS1) Gene in Broad Bean Plant

**Figure 4:**
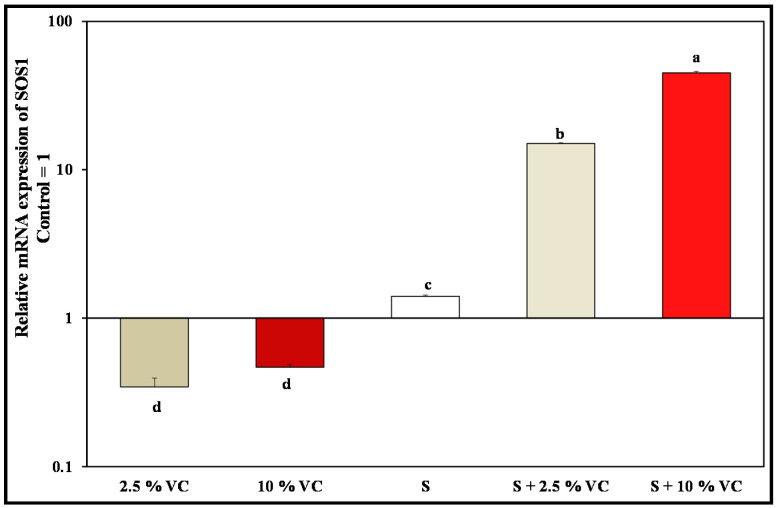
Changes in relative mRNA expression of SOS1 in leaves of broad bean plants treated with 2.5, 10% VC, 200 mM NaCl (S) and 200 mM NaCl combined with 2.5 or 10% VC after 30 days of cultivation. The values reported in the figure are means ± standard deviation of three replicates. Different letters on the bars indicates significant differences among treatments as evaluated by Duncan’s multiple comparison test (*p* < 0.05).

Salt Overly Sensitive (SOS1) gene comprises one of three components of the SOS signaling pathway in plants, which expels Na^+^ out of the cytoplasm to the external medium. In the current work, gene expression of SOS1 in broad bean plants leaves exhibited down-regulation at 2.5 and 10% VC by about 0.34 and 0.47-fold, respectively (Figure 4). Then, a gradual significant up-regulation was attained under 200 mM NaCl in addition to 2.5% VC and 200 mM NaCl by about 1.4 and 15-fold, respectively, over control. Interestingly, the highest significant up-regulation of SOS1 gene expression by about 45-fold over control is observed in leaves grown under the interaction between 10% VC and 200 mM NaCl.

## 3. Discussion

Agricultural soil affected by salinity is a matter of concern in many countries [14]. Among the different strategies, earthworm-mediated vermitechnology remains one of the best for the amelioration of salt stress and the enhancement of crop production [13].

The present study investigated the potential role of vermicompost in alleviating salt toxicity in broad bean plants by assessing various parameters related to their morphological traits, K^+^/Na^+^ homeostasis, osmotic adjustment, antioxidant system and SOS1 gene expression under saline conditions. Our results showed that vermicompost have successfully ameliorated salinity-induced damage, thereby offering a cost-effective salt stress management strategy during broad bean cultivation in saline soils.

Salinity disturbs crop growth by specific ion toxicity, osmotic stress, and nutrient imbalances [15]. The eventual effects of salinity on plant performance can be viewed as growth inhibition, leading to biomass reduction, as it was assessed in this study in terms of reduction of plant height, and fresh weight of broad bean plants grown under saline conditions. It was found that in *Triticum durum*, cultivar shoot length STI and shoot fresh weight STI were reduced in the presence of two salinity levels [16]. Indeed, a gradual increase in STI of all morphological traits with vermicompost treatments was noted, with the highest increase at 10% VC under salt stress. Interestingly, vermicompost is known to enhance plant growth through the stimulation of auxin (IAA), promoting soil beneficial microbes which induce plant growth, either directly through the production of plant growth regulating hormones and enzymes, or indirectly by managing plant pathogens, improving plant health, and minimizing yield loss [17]. Similar results in improving plant growth using vermicompost is reported by Nandi et al. [18] in pomegranate and by Ayyobi et al. [19] in peppermint plants.

Membership function value (MFV) was also used to evaluate salt tolerance where an increase in mean MFV indicates higher salt tolerance of broad bean plants. The results clarified that after the ranking of mean MFV, three levels of salinity tolerance were obtained: RSS at salt treatment, together with 2.5% VC and S; RST at 5% and 15% VC under salt treatment and RHST at 10% VC with salt stress. Consequently, it is confirmed that 10% VC of salt-stressed plants can effectively improve and discriminate morphological growth traits to the extent that the same cultivar may perform in a variable manner, depending on the VC% used. Moreover, it was validated that the valuable constituent of VC improves elemental status and regulates protein expression at transcriptional and translational levels, which may stabilize photosynthetic apparatus and manage the salt-induced harmful impacts that have been expressed on morphological traits in such an economically important plant as broad bean [20].

Under the prevailing experimental conditions, two distinct patterns of morphological parameters were observed; one of them was at 2.5 and 5% VC which was found to be very close, whereas the other observed above 10% VC with a decreasing trend. This could be explained by the fact that VC contains various elements and a lot of variable microorganisms, in addition to hormone-like substances that enhance plant growth at VC levels lower than or equal to 10%, while 15% VC showed that overdoses lowered its positive effect. Concurrently, it was found that higher VC proportions led to a lower positive effect, or even a disappearance of the significant positive effect on the root biomass of several plants [21].

Salt-induced toxicity (Na^+^) causes a reduction in mineral elements, including K^+^, Ca^2+^ and Mg^2+^, causing impairment of cellular metabolism; and thus, poor performance of salt-exposed plants [22]. In the present study, salt-treated broad bean plants exhibited a remarkable accumulation of Na^+^ in both roots and shoots, clearly explaining the association of ion toxicity with the deleterious effects of salt on the biomass of broad bean plants. Moreover, Na^+^ toxicity often results in K^+^ deficiency and affects K^+^/Na^+^ ratio [23], as was also observed in this study. The shortage of K^+^ can exacerbate Na^+^ toxicity by hampering several key physiological processes, such as stomatal movement, photosynthetic performance, metabolism of secondary metabolites, maintenance of membrane potential and osmotic balance, water status, and enzyme activation [24]. The exogenous application of VC improved the uptake of K^+^, Ca^2+^ and Mg^2+^ across plant partitions. Under salinity stress, 10% VC can modify plasma membrane function, increase nutrient uptake and the assimilation of broad bean plant, as well as the translocation of photosynthates to the sink. The ability of VC to maintain the structure of the plasma membrane may have contributed to the significant decrease in Na^+^ content in roots and shoots. Ion balance of K^+^/Na^+^ is essential for plants and is one of the prerequisites of salt-tolerance [25]. Higher K^+^/Na^+^ together with Mg^2+^ content was recorded on salt-stressed *Solanum lycopersicum* L. supplemented with VCL [26]. In this connection, there was clear indication that VC improved the capability of plants to restrict Na^+^ uptake through roots, while maintaining a favorable ratio of K^+^/Na^+^ to compensate nutrient deficiency under salt stress due to the porous structure of VC [20]. Indeed, our PCA result also endorsed that 10% VC-treated broad bean plants under salinity stress had a positive correlation with root K^+^ content. The current work coincides with the idea that salt-tolerant varieties could keep favorable ion homeostasis through attaining better K^+^/Na^+^ ratio [22,27]. Moreover, VC-treated broad bean plants under salinity stress exhibited growth performance improvement, peculiarly in shoots by retaining high K^+^/Na^+^ ratio.

Magnesium, in addition to being the powerhouse behind photosynthesis enhancing root and shoot biomass, it decreases ROS and, thus, improves the plant defense system. The greater availability of Mg^2+^ in the presence of VC is consistent with the higher contribution of Mg^2+^ content in *Vicia faba*. These results coincided with Hernndez et al. [28] in *Lactuca sativa* and with Mukta et al. [29] in *Solanum lycopersicum*.

The obtained data mentioned that increasing calcium content under supplementation of 10% VC to salt stressed broad bean plant contributes by triggering SOS signal transduction keeping K^+^/Na^+^ ratio appropriate for better physiological performance of broad bean plant [30]. An elevation in Ca^2+^ concentration was recorded in leaves of *Phaseolus vulgaris* L. treated by salinity levels plus VC [31]. The increase in plant nutrient availability by earthworms is likely to be due to the ideal microenvironment of the earthworm gut [32]. Earthworms ingest plant growth promoting rhizospheric bacteria (PGPR) such as *Pseudomonas*, *Rhizobium*, *Bacillus*, *Azosprillium*, *Azotobacter*, etc. along with rhizospheric soil; they might become activated or increased during their passage through the earthworm digestive system [33]. This special group of PGPR enhances solubilization of nutrients, production of growth hormones and nitrogen fixation [34]. Conclusively, in the present study, it is to be stated that the best contribution of elements together with lower Na^+^ levels were achieved after application of 10% VC, which is a valuable result since the use of fertilizers with accumulated Na^+^ salts represent an important risk for soil salinization and plant toxicity.

Salinity decreased the contents of nitrogen, protein and IAA in broad bean plants. This consistent decrease in N concentration due to salinity may be associated with a reduction in nitrate absorption. Broad bean imbalance in N-uptake and its translocation to leaves; likewise, protein degradation that disrupt carbon metabolism, might be due to alterations in intracellular ion homeostasis [35]. Indole acetic acid homeostasis may be influenced by salinity due to variations in IAA metabolism and distribution [36]. Nonetheless, proline and ABA increased under salt stress. Proline is reported to be a source of nitrogen and carbon that is used for growth, pH adjustment of cytosol, a free radical scavenger, detoxifier, allow rapid stress recovery and maintaining enzymes structure in a cell [37]. The significant increase in ABA under salinity stress could stimulate stomatal closure, cause changes in gene expression, and induce adaptive physiological responses [38].

Application of vermicompost under salt stress boosted nitrogen, protein and IAA contents accompanied by a significant decrease in ABA and proline. Vermicompost retained specific mechanisms for salt stress mitigation and growth promotion of broad bean plants, which may be explained in the context of Hand et al. and Atiyeh et al. [39,40] as they described that *E. foetida* favored nitrification in VC of cow manure through rapid conversion of ammonium-nitrogen to nitrate-nitrogen with a 28-fold increase in nitrate-nitrogen compared to only 3-fold increase in conventional compost. It was also reported the importance of VC fertilizer (as nitrogen stabilizer) to *Cicer arietinum* L. where an increase in protein content is directly related to an increase in nitrogen uptake by plants [41]. Zoubida and Gherroucha [42] postulated that IAA regulates plant growth response and the cell signaling cascade of different crop plants, as well as diminishing salt stress noxious effects. During vermicomposting, earthworms degrade the soil, thereby increasing the surface area for microbial degradation, improve the availability of O_2_, increase soil porosity and infiltration of water, accelerate the rate of mineralization, and increase growth [43]. Some recent studies provided evidence that vermicompost contains substantial amounts of auxin, CKs, ABA and GAs [44]. Nevertheless, ABA and proline are decreased; this may be due to the beneficial effect of VC as green technology substituting traditional physiological mechanisms that utilize ABA and proline as osmoprotectants under salt-stress. The decrease in proline may be due to improvement of root elongation while osmotic potential can be kept low by the increase in the concentration of soluble proteins in the cell sap; thereby enabling broad bean to take up water and elongate against the rigidity of the cell wall under salt stress [43]. *isenia*.

Hydrogen peroxide, as well as MDA contents, increased in response to the addition of 200 mM NaCl to the growth media of broad bean plants. Overproduction of MDA is a good indicator for the cells that fail to sweep excess ROS under salinity stress [45]. On the other hand, VC can reverse the effect of salt-treated broad bean plants. Such inversion could be explained by the beneficial effect of VC, which causes the reduction in ROS and membrane injury in response to salt stress. Application of vermicompost to rice under drought stress alleviated some of the deleterious stress-related effects on plant development [46]. Using the optimum level of fertilizer with twice the amount of K^+^ on *Brassica napus* decreases the inhibitory effects of salinity stress and increases the membrane stability index [47]. Findings obtained by Kiran [48] indicated the beneficial effect of VC on stressed *Lactuca* *sativa* var. crispa by reducing ROS and membrane injury. Vermicompost maintain better membrane stability and consequently efficient membrane function under salinity stress.

Oxidative stress is associated with the over production of toxic ROS [48]. Plants combat the oxidative stress via enzymatic and non-enzymatic machinery [49]. In the current work, the battery enzymatic antioxidant activities of SOD, CAT and APX recorded a general increase under salinity-stress, whereas PPO activity decreased under the same conditions. These results suggest that salinity provoked oxidative damage to salt-exposed broad bean plants. Upon addition of VC to the stressed plants, the highest increase in activity of the tested enzymes except GR was achieved at 10% VC. This may be due to the important role of SOD in playing the first line in the plant defense mechanism, protecting tissue against superoxide radical which damages the biological structure of the membrane. As a result of SOD activity, superoxide anion radical converts to H_2_O_2_ and O_2._ Catalase and ascorbate peroxidase also appear to be the most helpful defense against noxious H_2_O_2_ formed. Considering this point of view, higher activity of SOD, CAT and APX decreased the level of ROS in the cell, therefore increasing the stability of the membranes and reducing lipid peroxidation [48]. This confirms the effectiveness of VC in enhancing antioxidant mechanisms in *Vicia faba* plant under salt-stress. In consistence, the PCA results of the current study revealed that 10% VC under salinity stress attained significant correlation with leaves SOD and CAT together with APX in roots and leaves.

Salt-stress reduced AA/DHA and GSH/GSSG ratios in addition to a decrease in phenolic content for roots and leaves of broad bean plants. On the other hand, the addition of VC to salt-stressed broad bean plants improved antioxidant power of AA/DHA, GSH/GSSG, together with the phenolic content, especially at 10% VC, contributing to the reduction in oxidative burden for better tolerance to salt stress. The relative decrease in GR activity at 10% VC under salt treatment was associated with a corresponding reduction in the regeneration of both GSH and GSSG redox metabolites, suggesting that other pathways for detoxification and scavenging of ROS might have been involved. However, GSH still maintains a relatively higher level than GSSG, which reflected better ratio of GSH/GSSG to effectively overcome the noxious effect of salinity stress and preserve the main physiological functions of the cell. The increase in PPO activity at 10% VC and salinity stress is due to its function as an antioxidant activity of phenolic compounds, where it acts as a reducing agent, protects photosynthetic process from stress, and regulates cell death [50].

Free radicals oxidative stress of plasma membrane constituents is controlled by the activity of AA and phenolic compounds [51,52]. Furthermore, phenolic compounds participate in limiting the fluidity of cellular membranes via provoking membrane peroxidation, and thus enhancing their stabilization [51]. Expectedly, the obtained results revealed that broad bean plants exposed to salt stress showed a higher degree of lipid peroxidation, as revealed by the raised levels of MDA in leaves, in consistence with the reduced contents of phenolic compounds and AA in leaves. On the other hand, vermicompost improved the antioxidant power of broad bean plants under salt stress by enhancing the levels of AA and total phenolic compounds, which was associated with improved oxidative stress protection due to a heightened antioxidant system. Ait-El-Mokhtar et al. [53] stated that compost nullified the negative impacts of NaCl stress in *Phoenix dactylifera* L. by increasing the ratios of AA/DHA and GSH/GSSG and implying a better ROS scavenging system by fueling the ASC–GSH cycle.

Hierarchical clustering, together with a heat map, attained an effective discrimination of the applied treatments into two groups: control, 2.5 and 10% VC in group A; whereas, S, 2.5% VC and S in addition to 10% VC and S in group B. Analysis of PCA declares that 2.5, 10% VC from group A; 2.5% VC and S in addition to 10% VC and S from group B constitute three associations of applied treatments and certain parameters. Unexpectedly, 10% VC and 200 mM NaCl achieved a peculiar group of parameters associated with the relatively highest trend in comparison to salt-stressed treatment (namely, Ca^+2^; APX in roots and leaves; together with roots K^+^ and leaves SOD; CAT). Herein, such associated parameters are related to attributes such as growth, enzymatic antioxidant, and potassium replacement of sodium ions by effluxing through the common K^+^/Na^+^ ion pump in collaboration with Ca^+2^ role in the SOS1 signaling pathway via the plasma membrane. Consistently, the exogenous application of vermicompost with saline stress improved plant growth, photosynthetic efficiency, and enzymatic antioxidant systems activity [54]. The recovery of antioxidant enzyme activity was due to vermicompost application which minimizes plasma membrane fluidity and Na^+^ ion intrusion to elicit salinity stress [55]. Additionally, the potential of calcium-fortified composted animal manure is estimated to significantly enhance growth and yield of *Brassica napus* L. causing maximum increase in nitrogen, phosphorus, and potassium concentrations in shoots [56].

Salt overly sensitive pathway (SOS1) is a Na^+^/H^+^ exchanger and well-reported salt of the cell to sweep out Na^+^ ions [57]. The SOS pathway comprises three components, SOS1, SOS2, and SOS3 [58]. SOS1 is a plasma membrane Na^+^/H^+^ channel protein antiporter with 10–12 transmembrane domains, which entails the export of Na^+^ out of the cytoplasm to external medium and may also be a Na^+^ sensor [59,60].

In this study, the considerable induction of SOS1 transcript under 200 mM NaCl by about 1.4-fold the control was found. Concurrently, the overexpression of the HtSOS1 gene in rice could exclude more Na^+^ and accumulate more K^+^ [61]. The expression level of GhSOS1, which was cloned from a salt-tolerant genotype of *Gossypium hirsutum*, was significantly upregulated at salinity stress [62]. The transcriptional levels of SOS1, SOS2, and SOS3 in *Arabidopsis thaliana* are significantly upregulated under salinity stress over time in the atbzip62 mutants, while they were downregulated in the wild type [57]. In contrast, The SlSOS1-silenced transgenic tomato plants leaves, and roots accumulated more Na^+^ [58]. The SOS1 mutant lines of *Thellungiella* *salsuginea* [63], *Physcomitrella* *patens* [64], *Medicago truncatula* and *Medicago falcate* [65] showed excessive Na^+^ accumulation in cells.

In this study, we also found a significant induction of SOS1 transcript under the under combined treatment 10% VC and 200 mM NaCl by about 45-fold over the control, which is correlated with lower Na^+^ content together with higher K^+^ in the shoot. This might be explained by the mechanism of stressed-broad bean plants to pump more Na^+^ out of the cell, either via the loading of Na^+^ into the xylem or via extrusion of Na^+^ back to the growth medium. This enhancement of SOS1 gene expression could improve salt tolerance of broad bean plant. It was evident by Song et al. [66] that there is a strong synergistic effect between vermicompost and PGPR on crop quality of tomato and spinach. PGPR-inoculated salt-stressed plants recorded 2-fold higher SOS1 expression with respect to the uninoculated salt-stressed wheat plants [67]. Interestingly, it was declared that soil supplemented with VC together with *Eisenia foetida* gut is inhabited by strains of *Bacillus subtilis* and *Azotobacter chrooccocum* [68]. These bacterial strains are characterized by tolerance to elevated concentration of NaCl (1–15%). They were also reported to improve plant growth and enhance the tolerance to sodium chloride in barley, maize, rice, soybean, sunflower, and wheat [69]. This effectively highlights the attenuating role of 10% VC in cooperation with microorganisms to 200 mM NaCl salinity stress study.

As previously mentioned, an increase in the Ca^2+^ content of broad bean plants was recorded at 10% VC and 200 mM NaCl, with respect to salt-stressed plants which leads to a decrease in Na^+^ content and improvement of K^+^/Na^+^ ratio. It means an increase in Ca^2+^ content which, when perceived by SOS3, is able to start the regulation mechanism of ion homeostasis via the SOS signaling pathway. It was reported that, SOS3 binds to the activated SOS2 [70]. The activated SOS3-SOS2 protein kinase complex can phosphorylate plasma membrane Na^+^/H^+^ antiporter SOS1 to effectively pump Na^+^ together with its deleterious effect out of the cell [71].

**Figure 5:**
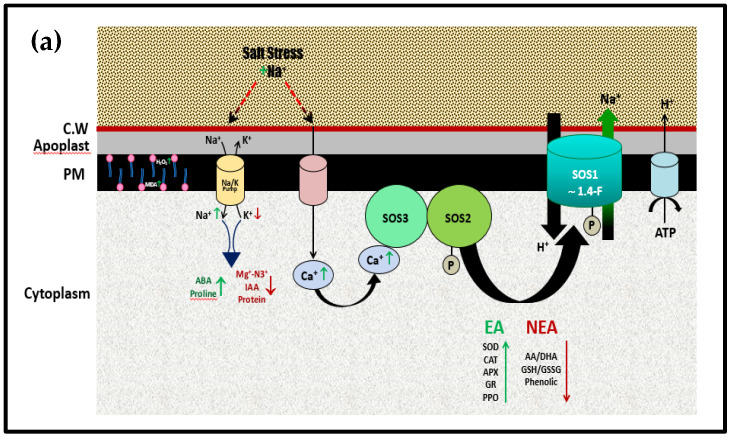

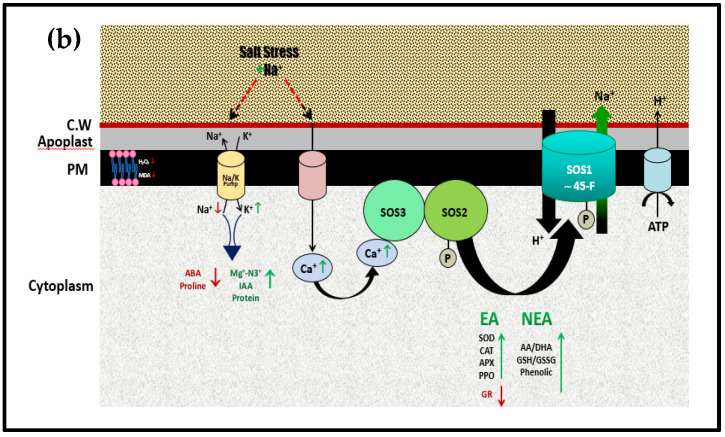
(**a**). Proposed regulation mechanism for the physiological role of SOS signaling pathway in response to 200 mM NaCl stress of *Vicia faba* plant leaves, adapted after [71]. CW: cell wall, PM: plasma membrane, EA: Enzymatic antioxidants; NEA: Non-Enzymatic antioxidants. (**b**). Proposed regulation mechanism for the physiological role of SOS signaling pathway in eliciting 200 mM NaCl stress of *Vicia faba* plant leaves previously supported with 10% VC, adapted after [71].

This is likely to summarize the effect of 10% VC under salinity stress on the molecular role of SOS1 in enhancing the salt tolerance of broad bean plants. The proposed illustrative mechanism of how SOS1 cooperates with SOS3/SOS2 complex through signal transduction pathway and interrelationship with other important physiological processes under salinity stress of *Vicia faba* leaves is represented in Figure 5a. The mechanistic alleviative role of 10% VC to salinity stress of NaCl and its effect on either SOS pathway or physiological traits is illustrated in Figure 5b.

## 4. Materials and Methods

### 4.1. Experimental Materials and Vermicompost Characterizations

A pure variety of broad bean plant seeds (*Vicia faba* L. Aspani) cultivar was obtained from Nubaseed Company. Uniform shape, size and viable seeds of broad bean were chosen, surface sterilized with 0.1% (*v*/*v*) HgCl_2_ solution for 5 min. and then rinsed three times with distilled water. Seeds were pre-soaked for 12 h before planting. Soil mixture composed of (Clay: sand; 1: 2) and vermicompost were used as the growth substrate. The soil mixture was characterized by electric conductivity (EC) 2.69 ± 0.01 uS cm^−1^, pH 7.71, N 0.109 ± 0.001%, P 0.048 ± 0.001% and K 0.349 ± 0.02%. Vermicompost used was obtained from the Agricultural Research Center, Giza, Egypt. Agricultural residues from rice straw and tree leaves were used as materials for preparation of VC in vermicomposting bins (100 × 120 × 50 cm), upon which the most common and productive earth worms “*Eisenia foetida*” was inoculated [72]. The product of this VC characterized by electric conductivity (EC) 6.5 uS cm^−^^1^, pH 6.56, total N 8.35 ± 0.06%, P 4.36 ± 0.06%, K 7.12 ± 0.06%, Ca 2.01 ± 0.02%, Mg 2.93 ± 0.04%, Cu 1.6 ± 0.09%, C 1.9 ± 0.07%, Silica 1.18 ± 0.17%, Mn 1.83 ± 0.08%, Na 1.48 ± 0.15%, B 0.03 ± 0.01%, Zn 2.8 ± 0.11%, Fe 0.99 ± 0.9%, Cl 0.03 ± 0.01%, SO_4_ 0.18 ± 0.02%, and Mo 0.05 ± 0.01%.

### 4.2. Experimental Design

Experiments of the current study were carried out in the Botanical Garden at the Faculty of Science, Alexandria University, Egypt. Five different ratios of dried vermicompost (0, 2.5, 5, 10, and 15%) were intermingled thoroughly with previously prepared soil mixture. The ratios including: 0:100; 2.5:97.5; 5:95; 10:90 and 15:85 and sublethal concentration of salt stress at 200 mM NaCl (the selected concentration of NaCl was determined after preliminary salinity screening from 50–400 mM NaCl); in addition to control [73]. The experiment was carried out under controlled conditions (Photon flux density (PFD) 375 µmol m^−2^ s^−1^, 10/14 h light/dark cycle, temperature 20 ± 2 °C and relative air humidity about 85%). After sowing, the field capacity of the culture pots was estimated by 200 mL demineralized water.

Experiments were carried out using a homogenous dried soil mixture in plastic culture pots (15 cm diameter and 15 cm height) with 1.25 kg soil capacity. In each pot and below the soil surface at 1 cm depth, six pre-soaked seeds of broad bean were located, where after emergence they were thinned to three seedlings per pot. The experiments were designed in the form of two sets using the selected five different volumetric ratios of VC and soil mixture. The first set of experiments involved fifteen pots, including the above-mentioned volumetric ratios irrigated with water. The second set of experiments consisted of fifteen pots set out exactly as the first set of experiments, but irrigated with 200 mM NaCl. The experiments were carried out as factorial Randomized Complete Block Design, with three replications.

All the pots were then regularly irrigated at field capacity with distilled water or 1/4 strength Hoagland solution (day after the day) throughout the experimental period. After 10 days of sowing, the first set was irrigated with demineralized water once and then again with 1/4 N Hoagland solution to supply plant nutrient requirements [74]. The second set was irrigated, once with 200 mM NaCl and again with 200 mM NaCl in 1/4 N Hoagland solution. Broad bean samples were harvested 30 days after planting, washed with running tap water, followed by demineralized water, then blotted gently using layers of tissue paper.

### 4.3. Measurement of Growth Traits

Root and shoot systems were separated and morphological traits via plant height (cm), fresh weight of root and shoot systems (g plant^−1^) were immediately determined. Dry weight samples were dried at 80 °C till a constant dry weight was reached. The salt-tolerance index (STI) for different treatments were calculated as the ratio of the value for the NaCl-treated plant/value for the control [75]. Salt-tolerance index of vermicompost-treated plants is the ratio of the value for the NaCl-treated plant under VC/value for its corresponding VC control; STI was calculated for all growth traits used.

Salt tolerance of broad bean was evaluated using the membership function value (MFV) according to Chen et al. [76]. The MFVof salt tolerance was calculated as follows:

Xi = (X − X_min_)/(X_max_ − X_min_) × 100%

where, Xi is the MFV of STI in a specific V %, X is the actual measured value of STI in a specific VC%, and X_max_ and X_min_ are the maximum and minimum values observed in all VC percentages, respectively [77]. Mean MFV is the average of MFVs of previously measured morphological traits; the bigger MFV mean the higher the salt tolerance.

### 4.4. Biochemical Analysis

#### 4.4.1. Elemental Analysis

Sample preparation, metal analysis and quality control were carried out according to the standard method of Kimbrough and Wakakuwa [78]. Oven-dried and homogenously milled 200 mg of plant samples were mixed with 3 mL of concentrated HNO_3_ in a beaker and covered with a ribbed watch glass. Then, the mixture was heated on a hot plate at 90–95 °C and left to evaporate to a low volume. After cooling, the previous step was repeated with additional portions (3 mL) of HNO_3_ until the digested solution either turned into a lighter color or reached a stable color, and the digestate was then refluxed with a small portion of HCl (3 mL) for complete digestion. Finally, the sample was filtered through filter paper (Whatman 42, diameter 110 mm). Then, the beaker walls and watch glass were washed with deionized water and the filter paper was rinsed with diluted HNO_3_ (10%). The final volume was adjusted to 25 mL with deionized water. To determine different metal contents, the solutions were subjected to Inductively Coupled Plasma-Optical Emission Spectroscopy (ICP-OES; Agilent 5100 VDV, Santa Clara, CA, USA). The content of Na^+^, K^+^, Ca^2+^ and Mg^2+^ were computed as parts per million (ppm). Flow rates of plasma, auxiliary and nebulizer of ICP-OES were kept at 12, 1, 0.7 mL min^−1^, respectively. The sample uptake and stabilization time were 10 s for each sample.

#### 4.4.2. Determination of Nitrogen

Estimation of the percentage of dry matter was prepared by well-cut shoot samples ranging from 30 to 50 g, then placed in an air-forced oven at a temperature of 70 °C for 72 h. The prepared dry samples were used to estimate percentage of nitrogen (N) content according to AOAC [79].

#### 4.4.3. High-Performance Liquid Chromatographic (HPLC) Method for the Determination of Abscisic Acid and Indole Acetic Acid

One gram of fresh tissue was homogenized with 70% (*v*/*v*) methanol and stirred overnight at 4 °C. The extract was filtered through a Whatman filter, and the methanol evaporated under vacuum. The aqueous phase was adjusted to pH 8.5 with 0.1 M phosphate buffer and then partitioned with ethyl acetate 3 times. After removal of the ethyl acetate phase, the pH of the aqueous phase was adjusted to 2.5 with (1N) HCl. The solution was partitioned with diethyl ether 3 times, and then passed through anhydrous sodium sulfate. After that the diethyl ether phase was evaporated under vacuum and the dry residue containing hormones was dissolved in 2.0 mL of methanol and stored in vials at 4 °C.

The separation procedure was obtained according to Kelen et al. [80] with some modifications with High-Performance Liquid Chromatography (HPLC) Agilent technology infinity 1260 (Germany) equipped with an Agilent Multiple wavelength ultraviolet detector (MWD). The flow rate was (1.2 mL min^−1^) of the mobile phase acetonitrile water (30:70% *v*/*v*) and a column temperature compartment was adjusted to 35 °C. Five microliters of each sample were injected onto the HPLC column using the autosampler apparatus with a 100 uL sample loop. Separation was performed on ZORBAX Eclipse Plus C18 (250 × 4.6 mm id, 5 um particle size analytical column. The signal of the compounds was monitored at 260 and 280 nm for, ABA and IAA (PhytoTech labs; Shawnee Mission, KS, USA), respectively. Data were managed using an HP ChemStation software.

#### 4.4.4. Determination of Free Proline

Fresh samples (100 mg) were ground with 3% sulfosalicylic acid and centrifuged at 4500× *g* for 10 min. The supernatant was decanted, and extract was adjusted to a known volume with 3% sulfosalicylic acid. Proline content was estimated by method described by Bates et al. [81]. Aliquot of 2 mL from plant extract was mixed with 2 mL of freshly prepared acid ninhydrin reagent and 2 mL of glacial acetic acid in a test tube. The mixture was incubated at 100 °C for 1 h, and then terminated the reaction by cooling the mixture in an ice bath. Add to mixture 4 mL of toluene and mixed by using a vortex for 20 s and then stand 10 min. The organic layer of colored toluene which containing the chromophore was separated to quantity the amount of proline. Concentrations of proline in the plant tissue was determined from a standard curve and expressed as µg g^−1^ FW.

#### 4.4.5. Estimation of Protein Content

Soluble proteins were extracted by adding 10 mL of distilled water to about 100 mg of the oven-dry plant material, then boiling for 5 min. After cooling, the extract was completed to volume (50 mL) with distilled water. This extract was used to estimate protein content according to Hartree [82]. One ml of the extract was mixed in a tube with 0.9 reagent A (two g potassium sodium acetate tartarate + 100 g sodium carbonate were dissolved in 1 litre of 0.1 N sodium hydroxide), and incubated in a water bath at 50 °C for 10 min. After cooling to room temperature, 0.1 mL of reagent B (two g potassium sodium acetate tartarate +1 g CuSO_4_·5H_2_O were dissolved in 100 mL of 0.1 N sodium hydroxide) was added, mixed well and the tubes were allowed to stand for 10 min. Three ml of reagent C (one volume Folin-Ciocalteau reagent, diluted to ten volumes with distilled water) were rapidly added with mixing, using a vortex mixer. The tubes were then incubated at 50 °C for 10 min. After cooling, the color was measured at 650 nm and the concentration was read from standard curve made with bovine serum albumin and expressed as mg g^−1^ DW.

#### 4.4.6. Determination of Endogenous MDA and H2O2 Content

Malondialdehyde (MDA) as the product of the lipid peroxidation reaction was determined by the method described by Heath and Paker [83]. Fresh plant samples (0.2 g) were homogenized in 5 mL 0.1% (*w*/*v*) trichloroacetic acid (TCA) solution on ice bath. The homogenates were centrifuged at 12,000 ×*g* for 5 min at 4 °C and the supernatants were collected in clean test tubes. The reaction mixture contained 0.5 mL aliquot of plant extract and 1 mL of 20% (*w*/*v*) TCA containing 0.5% (*w*/*v*) thiobarbituric acid (TBA). The mixture was kept in a boiling water bath for 30 min and cooled immediately on ice. After centrifugation at 10,000 ×*g* for 10 min, the non-specific absorbance of the supernatant at 600 nm was subtracted from the maximum absorbance at 532 nm for MDA measurement. The concentration of MDA was calculated using an extinction coefficient of 155 mmol L^−1^ cm^−1^.

Hydrogen peroxide content was determined according to Velikova et al. [84]; 0.2 g of plant tissues (leaves or roots) were homogenized in an ice bath with 5 mL of cold 0.1% (*w*/*v*) trichloroacetic acid (TCA). The homogenate was centrifuged at 12,000× *g* for 15 min. 0.5 mL of the plant extract was added to 0.5 mL of 10 mM potassium phosphate buffer (pH 7) and 1 mL of 1 M KI. The absorbance of the supernatant was measured at 390 nm. The content of H_2_O_2_ was calculated by comparison with a standard calibration curve and the results were expressed as µmol H_2_O_2_ g ^1^ FW.

#### 4.4.7. Enzyme’s Extractions and Assays

Two gram of fresh broad bean leaves and roots were ground to a fine powder in liquid N_2_ and then homogenized in 2 mL of 50 mM potassium phosphate buffer (pH 7.0), 1 mM EDTA, 1mM D-isoascorbic acid, 2% (*w*/*v*) poly-vinyl pyrrolidone (PVP) and 0.05% (*w*/*v*) Triton X-100 using a chilled pestle and mortar following the method of Gossett et al. [85]. The homogenate was centrifuged at 10,000× *g* for 10 min at 4 °C and the supernatants were collected and used for the assays of catalase, ascorbate peroxidase, glutathione reductase and polyphenol oxidase.

- Estimation of catalase (EC 1.11.1.6) activity;

Catalase (CAT) activity was assayed spectrophotometrically by measuring the rate of H_2_O_2_ disappearance at 240 nm, taking extinction coefficient (Δε) 2.8 mM^−1^ cm^−1^ as 43.6 mM^−1^ cm^−1^. The reaction mixture contained 50 mM potassium phosphate (pH 7.0), 10.5 mM H_2_O_2_ [86]. The reaction started at 25 °C for 2 min, after adding of 100 μL the enzyme extract, and the initial linear rate of decrease in absorbance at 240 nm was used to calculate the activity.

- Estimation of ascorbate peroxidase (APX, EC 1.11.1.11) activity

Ascorbate peroxidase (APX) was assayed as described by Nakano and Asada [87]. The reaction mixture contained 50 mM potassium phosphate (pH 7.0), 0.2 mM EDTA, 0.5 mM ascorbic acid and 0.25 mM H_2_O_2_. The reaction was started at 25 °C by the addition of H_2_O_2_ after adding the enzyme extract containing 50 μg of protein. The decrease in absorbance at 290 nm for 1 min was recorded and the amount of ascorbate oxidized was calculated from the extinction coefficient (Δε) 2.8 mM^−1^ cm^−1^.

- Estimation of glutathione reductase (EC 1.6.4.2) activity

Glutathione activity was determined according to Smith et al. [88]. The reaction mixture contained 1.0 mL 0.2 M potassium phosphate (pH 7.5) containing 1 mM EDTA, 0.5 mL 3 mM DTNB in 0.01 M phosphate buffer, 0.25 mL H_2_O, 0.1 mL 2 mM NADPH, 0.1 mL enzyme extract, 0.1 mL 20 mM GSSG. The reaction initiated by the addition of GSSG. The temperature was maintained at 24 °C, the increase in absorbance at 412 nm was monitored for 5 min.

- Estimation of superoxide dismutase (SOD, EC 1.15.1.1) activity

For the assay of SOD, superoxide dismutase (SOD) was homogenized in 8 mL potassium phosphate buffer (50 mM, pH 7.8) containing 0.1 mM Na_2_EDTA and 1% insoluble polyvinyl pyrrolidone PVP with a chilled pestle and mortar. The homogenate was centrifuged at 20,000× *g* for 20 min. The supernatant was collected and used for the assay of SOD following the method of Beyer and Fridrovich [89]. The reaction mixture was prepared by mixing 27 mL of 50 mM potassium phosphate, pH 7.8, 1.5 mL of L-methionine (300 mg 10 mL^−1^), 1 mL of nitrobluetetrazolium salt (14.4 mg 10 mL^−1^), and 0.75 mL of Triton X-100. Aliquots (1 mL) of this mixture were delivered into small glass tubes, followed by 20 μL of enzyme extract and 10 μL of riboflavin (4.4 mg 100 mL ^1^). The cocktail was mixed and then illuminated for 7 min in an aluminum foil-lined box, containing two 20 W fluorescent tubes. A control tube in which the sample was replaced by 20 μL of buffer was run in parallel and the A560 was measured in all tubes. The increase in absorbance due to formazan formation was read at 560 nm. Under the described conditions, the increase in absorbance without the enzyme extract was taken as 100% and the enzyme activity was calculated by determining the percentage inhibition per min. Fifty percent of inhibition was taken as equivalent to 1 unit of SOD activity.

- Estimation of poly phenol oxidase (PPO, EC 1.10.3.)

The assay activity of PPO using a spectrophotometric method was based on an initial rate of increase in absorbance at 410 nm [90]. Phosphate buffer solution pH 7 (0.1 M, 1.95 mL), 1 mL of 0.1 M catechol as a substrate and 100 µL of the enzyme extract were pipetted into a test tube and mixed thoroughly. Then, the mixture was rapidly transferred to a 1 cm path length cuvette. The absorbance at 410 nm was recorded continuously at 25 °C for 5 min at room temperature.

#### 4.4.8. Non-Enzymatic Antioxidant Extractions and Assays

Plant tissues (0.2 g) were homogenized in 5 mL of ice-cold 5% meta-phosphoric acid and centrifugation at 12,000× *g* for 10 min at 4 °C. The supernatant was collected for the determination of water-soluble antioxidant contents (ascorbate and glutathione content).

- Estimation of ascorbic acid content

Ascorbic acid (AA) and dehydro-ascorbic acid (DHA) were determined as described by DePinto et al. [91]. This analysis is based on the reduction from Fe^3+^ to Fe^2+^ of the ascorbic acid (ASC), followed by the spectrofotometric determination of ion Fe^2+^ complexed with 2,2-dipiridil. Dehydroascorbic acid (DHA) is reduced to AA through incubation with dithiothreitol (DDT). Excess of DDT is removed with N-ethylmaleimide (NEM) and total AA is determined using 2,2 bipiridil method. 300 μL of supernatant was used for the AA assay and 750 μL potassium phosphate buffer (100 mM, pH 7.2) and 300 μL distilled water were added to the extract. 300 μL of the supernatant was used for the DHA assay and 750 μL potassium phosphate (100 mM, pH 7.2) and 150 μL of dithiotheritol (10 mM) were added. The samples were incubated at room temperature for 10 min, and then 150 μL of 0.5% N-ethylmaleimide was added. Both samples were vortexed and incubated at room temperature for 10 min. To each sample 600 μL of 10% (*w*/*v*) TCA, 600 μL of 44% (*v*/*v*) orthophosphoric acid, 600 μL of 4% (*w*/*v*) bipyridyl in 70% (*v*/*v*) ethanol and 10 μL of 3% FeCl_3_ were added. After vortex-mixing, samples were incubated at 40 °C in a water bath for 20 min, and then samples were vortexed again and incubated at 40 °C in a water bath for another 20 min. The absorbance of samples was recorded at 525 nm. A standard curve of AA and DHA was used for the calculation of AA and DHA concentration. Concentration of DHA is calculated by difference between total AA and reduced form of AA (without treatment of DDT).

- Estimation of glutathione content

Reduced glutathione and oxidized glutathione (GSSG) levels were determined according to the method of Griffith [92]. Tissues from control and treated plants were ground using a pestle and mortar in liquid N_2_ with 1 mL of 5% sulpho-salicylic acid and centrifuged at 10,000× *g* for 5 min. A 300 mL aliquot of the supernatant was removed and neutralized to pH 7.0 by adding l8 µL of 7.5 mM triethanolamine. A 150 mL sample was used for the determination of total glutathione (GSH+ GSSG). Another 150 µL sample was pre-treated with 3 mL 2- vinyl pyridine for 60 min at 28 °C to mask GSH and to allow the determination of GSSG alone. Fifty micro liters from both types of samples were mixed with 700 µL of 0.3 mM NADPH, 100 µL of 10 mM 5,5′-dithiobis-(2-nitrobenzoic acid) (DTNB), and 150 µL of 125 mM NaPO4–6.3 mM EDTA buffer, pH 6.5. Ten micro liters of glutathione reductase (GR) (50 U/mL) was added and the change in absorbance at 412 nm was monitored after 5 min.

- Extraction and determination of total phenolic content

Total phenolic content of extracted samples was determined according to Folin-Ciocalteau spectrophotometric method of Singleton and Rossi [93] with modification. 200 µL of extract was placed in a reaction test tube to 1 mL of water and 100 µL of Folin-Ciocalteau reagent (Sigma) were added. The test tubes were allowed to stand for 10 min, and then 200 µL of 20% NaCO_3_ was added. After 20 min at 40 °C, the absorbance was measured at 750 nm. Phenolic concentration was calculated according to a calibration curve using gallic acid as a standard and the results were expressed as mg gallic acid g ^1^ FW. 

### 4.5. Gene Expression Analysis of SOS1

Frozen leaf tissues (–80 °C) were thawed on ice and 100 mg of frozen sample were used for RNA extraction. The concentration of RNA sample was determined using NanoDrop spectrophotometer (ThermoFisher Scientific, USA). To synthesize the first complementary strand DNA (cDNA), 500 ng of each RNA sample was reverse transcribed using AMV reverse transcriptase system (Promega, USA). A degenerate pair of primers, corresponding to sequences of the conserved amino acidic regions of the plant SOS1 gene was used. Forward primer for actin was: 5′GAGACTTTCAATGCCCCTGC3′, whereas the reverse primer was: 3′CCATCTCCAGAGTCGAGCACA5′. For a gene of interest, the qRT-PCR reaction was carried out in triplicates. The forward primer for SOS1 was: 5′-GC(A/T)TGCAT(G/C)A(G/C)TT(C/) TGGGAAATG-3′ while the reverse primer was: 5′-GTCTGGACAGCAT (A/C)(A/G)TGAAGATG-3′ [58]. The thermal cycling was performed as initial pre-heating step at 95 °C for 10 min; 40 cycles of denaturing at 95 °C for 5 s, annealing at 60 °C for 30 s and extension at 72 °C for 30 s using qRT-PCR (ViiA™ 7, Applied Biosystems^®^, ThermoFisher Scientific, USA). The relative gene expression was calculated as follows: 2-ΔΔCT = normalized gene expression. Normalizing the expression of the target gene to that of the reference gene compensates for any difference in the amount of the sample tissue [94].

### 4.6. Statistical Analysis

Obtained data were analyzed using one-way analysis of variance (ANOVA) approach. Duncan’s multiple comparison range tests using SPSS software [95] was carried out to identify statistically significant differences among the treatments at *p* < 0.05. Data were presented as means ± standard deviation (*n* = 3), and different alphabetical letters revealed significant differences among the treatments. Statistical analysis software followed the methods of Sokal and Rohlf [96].

Data was analyzed using the two-way cluster analysis and principal component analysis (PCA) using PC-ORD ver. 6. Two-Way cluster analysis has been conducted for the purpose of clustering control and treatment traits. Euclidean distance and Ward’s linkage method were used to achieve the task. A heat map with two cluster analyses were produced for the traits and for the parameters. Parameter’s rank is represented in color, where the intense red color represents the high value, while the light red color represents the low value. Principle component analysis (PCA) (linear ordination technique) has been conducted to find correlations among parameters in relation to different groups. Data was represented along the first two axes with a cumulative percentage of variance of around 89%.

## 5. Conclusions

The current results strongly promoted the usage of 2.5% VC-treated *Vicia* *faba* cultivar as an effective biofertilizer enabling seedlings of broad beans to perform better under normal conditions through improving growth and physiological parameters. The subjection of 10% VC growing media of *Vicia faba* seedlings to salt-stress declared that the mechanism of the SOS signaling pathway mediated K^+^/Na^+^ pump together with an increased Ca^2+^ influx via the plasma membrane. This enhanced the K^+^/Na^+^ ratio, allowing an increase in Mg^2+^, N, protein, and IAA contents in parallel with the inhibition of ABA and proline contents as stress responsive osmolytes. The powerful efflux of Na^+^ ions mediated by SOS1 via SOS signal transduction permit higher activity of all enzymatic (except GR) and non-enzymatic antioxidants. Higher activity of SOD, CAT and APX decreased the level of ROS, therefore reduced lipid peroxidation, decreased the fluidity of PM and thus the stability of membranes increased. It is to be stated that vermicompost-induction of SOS1 gene expression to about 45-fold the control validated the efficient role of SOS1 in triggering all the defense systems of the *Vicia faba* plant to cooperate for salinity-stress recovery, i.e., defense machinery is mediated by the SOS signaling pathway, beginning with Ca^2+^ sensor protein and ending with SOS1 effluxing Na^+^.

Research concerning the molecular behavior of the SOS signaling pathway with vermicompost under salinity stress in different plant varieties with economic importance is encouraged. Since vermicompost is highly rich in microbes; further research has to focus on the role of PGPR in ameliorating the current salinity problems of economic crops. Finally, different plant growth hormones are extracted from VC; thus, more investigations are expectant to elucidate the relation between hormone biosynthesis in VC and the plant root system.

## Figures and Tables

**Table 1:** Salt tolerance indices (STI) of plant height (PH), root fresh weight (RFW), shoot fresh weight (SFW), total fresh weight (TFW), root dry weight (RDW), shoot dry weight (SDW) and total dry weight (TDW) under 200 mM NaCl (S), 2.5, 5, 10 and 15%VC treatments of *Vicia faba* plant after 30 days of cultivation. Values are means ± SD based on triplicate independent determinations, and different letters means significant difference as evaluated by Duncan’s multiple comparison test (*p* < 0.05).

STI of Parameters	S	2.5% VC + S	5% VC + S	10% VC + S	15% VC + S
PH	0.33 ^bc^ ±0.06	0.31 ^c^ ± 0.03	0.38 ^bc^ ±0.03	0.55 ^a^ ± 0.08	0.45 ^ab^ ± 0.09
RFW	0.31 ^b^ ± 0.10	0.30 ^b^ ± 0.08	0.54 ^a^ ± 0.07	0.62 ^a^ ± 0.01	0.51 ^a^ ± 0.09
SFW	0.32 ^c^ ± 0.05	0.31 ^c^ ± 0.04	0.52 ^b^ ± 0.02	0.77 ^a^ ± 0.05	0.62 ^b^ ± 0.11
TFW	0.32 ^c^ ± 0.06	0.31 ^c^ ± 0.05	0.53 ^b^ ± 0.02	0.72 ^a^ ± 0.03	0.58 ^b^ ± 0.06
RDW	0.39 ^c^ ± 0.09	0.34 ^c^ ± 0.05	0.68 ^b^ ± 0.07	1.10 ^a^ ± 0.05	0.80 ^b^ ± 0.10
SDW	0.36 ^c^ ± 0.07	0.37 ^c^ ± 0.08	0.59 ^b^ ± 0.12	0.81 ^a^ ± 0.06	0.61 ^b^ ± 0.04
TDW	0.37 ^c^ ± 0.07	0.36 ^c^ ± 0.07	0.61 ^b^ ± 0.11	0.87 ^a^ ± 0.04	0.65 ^b^ ± 0.02

**Table 2:** Morphology results declared that 2.5 and 10% VC could be selected to test their role in amelioration of salinity stress. Table 2. Membership function value (MFV), ranking of MFV means of plant height (PH), root fresh weight (RFW), shoot fresh weight (SFW), total fresh weight (TFW), root dry weight (RDW), shoot dry weight (SDW) and total dry weight (TDW) under 200 mM NaCl (S), 2.5, 5, 10 and 15%VC treatments of *Vicia faba* plant after 30 days of cultivation. Values are means ± SD based on triplicate independent determinations, and different letters means significant difference as evaluated by Duncan’s multiple comparison test (*p* < 0.05).

MFV of Parameters	S	2.5% VC + S	5% VC + S	10% VC + S	15% VC + S
PH	18.5 ^bc^ ± 18.5	12.9 ^c^ ± 8.5	33.0 ^bc^ ± 8.1	80.5 ^a^ ± 23.7	53.4 ^ab^ ± 27.2
RFW	26.1 ^b^± 22.6	25.1 ^b^ ± 17.7	80.3 ^a^ ± 15.7	97.2 ^a^ ± 2.4	69.2 ^a^ ± 19.2
SFW	9.8 ^c^ ± 8.1	7.2 ^c^ ± 7.2	45.5 ^b^ ± 4.3	89.7 ^a^ ± 9.1	62.4 ^b^ ± 20.0
TFW	10.4 ^c^ ± 11.2	8.0 ^c^ ± 10.4	53.3 ^b^ ± 4.5	92.5 ^a^ ± 6.6	64.5 ^b^ ± 13.1
RDW	11.2 ^c^ ± 10.8	5.1 ^c^ ± 6.1	45.3 ^b^ ± 7.8	94.7 ^a^ ± 5.6	59.6 ^b^ ± 11.9
SDW	12.9 ^c^ ± 12.1	14.5 ^c^ ± 14.8	52.5 ^b^ ± 21.5	91.0 ^a^ ± 10.7	56.2 ^b^ ± 6.9
TDW	12.5 ^c^ ± 12.1	11.4 ^c^ ± 12.2	51.9 ^b^ ± 17.8	94.8 ^a^ ± 6.3	58.9 ^b^ ± 2.5
MFV	14.5 ^c^ ± 9.7	12.0 ^c^ ± 10.3	51.7 ^b^ ± 8.8	91.5 ^a^ ± 6.1	60.6 ^b^ ± 4.3
Ranking of MFV means	3	3	2	1	2

**Table 3:** Effect of 0, 2.5 and 10% vermicompost, 200 mM NaCl (S) and their interactions on the contents of calcium (Ca^2+^), magnesium (Mg^2+^), and potassium/sodium ratio (K^+^/Na^+^) in the roots and shoots of *Vicia faba* plant after 30 days of cultivation. Values are means ± SD based on triplicate independent determinations, and different letters means significant difference as evaluated by Duncan’s multiple comparison test (*p* < 0.05).

	Plant Tissues	Control	2.5%VC	10%VC	S	2.5%VC + S	10%VC + S
Mg^2+^ (g 100 g^−1^ DW)	Roots	0.82 ^ab^ ± 0.05	0.92 ^a^ ± 0.04	0.76 ^bc^ ± 0.06	0.60 ^d^ ± 0.11	0.65 ^cd^ ± 0.08	0.75 ^bc^ ± 0.11
	Shoots	0.36 ^ab^ ± 0.03	0.44 ^a^ ± 0.09	0.40 ^ab^ ± 0.05	0.22 ^c^ ± 0.05	0.25 ^c^ ± 0.06	0.30 ^bc^ ± 0.05
K^+^/Na^+^	Roots	1.22 ^c^ ± 0.04	1.46 ^a^ ± 0.01	1.28 ^b^ ± 0.01	0.36 ^f^ ± 0.01	0.69 ^e^ ± 0.01	0.81 ^d^ ± 0.01
	Shoots	4.45 ^c^ ± 0.30	6.63 ^a^ ± 0.42	5.22 ^b^ ± 0.42	2.32 ^e^ ± 0.10	3.26 ^d^ ± 0.15	4.39 ^c^ ± 0.45
Ca^2+^ (g 100 g^−1^ DW)	Roots	1.81 ^e^ ± 0.10	2.37 ^bc^ ± 0.04	2.22 ^cd^ ± 0.05	2.10 ^d^ ± 0.11	2.51 ^b^ ± 0.13	2.96 ^a^ ± 0.09
	Shoots	1.07 ^e^ ± 0.04	1.29 ^cd^ ± 0.08	1.17 ^de^ ± 0.05	1.41 ^c^ ± 0.08	1.77 ^b^ ± 0.05	2.12 ^a^ ± 0.11

**Table 4:** Effect of 0, 2.5 and 10% vermicompost, 200 mM NaCl (S) and their interactions on the contents of indole acetic acid (IAA), abscisic acid (ABA), proline, nitrogen (N) and protein in the roots and shoots of *Vicia faba* plant after 30 days of cultivation. Values are means ± SD based on triplicate independent determinations, and different letters means significant difference as evaluated by Duncan’s multiple comparison test (*p* < 0.05).

Parameters	Plant Tissues	Control	2.5%VC	10% VC	S	2.5% VC + S	10%VC + S
IAA (ng g^−1^ FW)	Roots	246.1 ^c^ ± 5.37	252.8 ^bc^ ± 5.16	265.9 ^a^ ± 6.31	153.4 ^e^ ± 3.80	173.3 ^d^ ± 3.97	260.4 ^ab^ ± 0.0
	Leaves	176.4 ^b^ ± 11.17	188.9 ^b^ ± 8.11	247.6 ^a^ ± 6.71	95.2 ^d^ ± 3.72	136.1 ^c^ ± 6.44	240.1 ^a^ ± 8.53
ABA (ng g^−1^ FW)	Roots	22.65 ^e^ ± 5.31	29.50 ^e^ ± 5.31	54.40 ^d^ ± 5.31	324.7 ^a^ ± 5.31	281.6 ^b^ ± 5.31	82.04 ^c^ ± 5.31
	Leaves	60.86 ^e^ ± 3.89	85.75 ^d^ ± 3.89	84.34 ^d^ ± 3.89	294.1 ^a^ ± 3.89	160.7 ^b^ ± 3.89	113.8 ^c^ ± 3.89
Proline (µg g^−1^ FW)	Roots	0.15 ^d^ ± 0.02	0.16 ^d^ ± 0.02	0.34 ^b^ ± 0.01	0.64 ^a^ ± 0.03	0.38 ^b^ ± 0.02	0.26 ^c^ ± 0.03
	Leaves	0.14 ^d^ ± 0.03	0.19 ^d^ ± 0.09	0.40 ^c^ ± 0.04	0.85 ^a^ ± 0.02	0.54 ^b^ ± 0.04	0.36 ^c^ ± 0.01
N (mg g^−1^ DW)	Roots	27.17 ^a^ ± 0.72	27.27 ^a^ ± 0.67	26.64 ^a^ ± 1.67	21.54 ^b^ ± 0.49	22.12 ^b^ ± 0.95	23.27 ^b^ ± 0.53
	Shoots	27.07 ^a^ ± 0.94	28.92 ^a^ ± 1.91	27.46 ^a^ ± 0.53	22.25 ^c^ ± 0.72	22.93 ^bc^ ± 0.85	24.73 ^b^ ± 1.75
Protein (mg g^−1^ DW)	Roots	170.0 ^a^ ± 6.08	172.0 ^a^ ± 2.65	165.0 ^a^ ± 5.57	135.0 ^c^ ± 4.36	139.0 ^c^ ± 3.61	147.0 ^b^ ± 3.46
	Shoots	176.0 ^b^ ± 2.65	191.0 ^a^ ± 2.65	171.0 ^b^ ± 3.61	137.0 ^d^ ± 1.73	141.0 ^d^ ± 3.46	155.0 ^c^ ± 3.61

**Table 5:** Effect of 0, 2.5 and 10% vermicompost, 200 mM NaCl (S) and their interactions on the contents of ascorbate (AA), dehydroascorbate (DHA), AA/DHA, reduced glutathione (GSH), oxidized glutathione (GSSG), GSH/GSSG, and phenolics in the roots and shoots of broad bean plants after 30 days of cultivation. Values are means ± SD based on triplicate independent determinations, and different letters means significant difference as evaluated by Duncan’s multiple comparison test (*p* < 0.05).

Parameters	Plant Tissues	Control	2.5%VC	10% VC	S	2.5% VC + S	10%VC + S
AA (nmol g^−1^ FW)	Roots	10.10 ^b^ ± 0.62	11.20 ^b^ ± 1.91	12.70 ^b^ ± 1.14	44.80 ^a^ ± 1.41	46.20 ^a^ ± 2.60	48.0 ^a^ ± 2.46
	Leaves	14.80 ^b^ ± 0.85	16.90 ^b^ ± 2.91	18.50 ^b^ ± 1.49	76.80 ^a^ ± 1.59	78.40 ^a^ ± 4.18	81.40 ^a^ ± 4.28
DHA (nmol g^-1^ FW)	Roots	8.80 ^b^ ± 0.62	9.10 ^b^ ± 1.91	10.80 ^b^ ± 1.14	42.20 ^a^ ± 1.54	41.60 ^a^ ± 2.60	41.0 ^a^ ± 2.91
	Leaves	9.0 ^c^ ± 0.85	9.60 ^c^ ± 2.17	11.0 ^c^ ± 1.49	75.80 ^a^ ± 1.59	65.80 ^b^ ± 4.18	66.40 ^b^ ± 4.37
AA/DHA	Roots	1.15 ^b^ ± 0.01	1.24 ^a^ ± 0.04	1.18 ^b^ ± 0.02	1.06 ^d^ ± 0.01	1.11 ^c^ ± 0.01	1.17 ^b^ ± 0.03
	Leaves	1.64 ^b^ ± 0.06	1.77 ^a^ ± 0.10	1.69 ^ab^ ± 0.09	1.01 ^d^ ± 0.0	1.19 ^c^ ± 0.02	1.23 ^c^ ± 0.02
GSH (nmol g^−1^ FW)	Roots	25.0 ^bc^ ± 2.31	24.50 ^bc^ ± 2.44	23.0 ^c^ ± 2.95	28.90 ^a^ ± 0.82	27.20 ^ab^ ± 1.22	26.50 ^abc^ ± 0.89
	Leaves	32.90 ^bc^ ± 1.15	29.20 ^cd^ ± 1.78	26.80 ^d^ ± 1.56	38.50 ^a^ ± 2.29	33.90 ^b^ ± 2.71	30.90 ^bc^ ± 2.70
GSSG (nmol g^−1^ FW)	Roots	19.80 ^c^ ± 2.51	18.90 ^c^ ± 2.79	18.0 ^c^ ± 2.42	26.60 ^a^ ± 1.31	24.40 ^ab^ ± 1.56	21.80 ^bc^ ± 0.62
	Leaves	23.20 ^bc^ ± 1.15	18.10 ^d^ ± 2.01	18.0 ^d^ ± 1.40	34.40 ^a^ ± 2.80	25.30 ^b^ ± 2.55	19.70 ^cd^ ± 2.0
GSH/GSSG	Roots	1.27 ^ab^ ± 0.05	1.30 ^a^ ± 0.08	1.28 ^ab^ ± 0.01	1.09 ^c^ ± 0.03	1.11 ^c^ ± 0.02	1.21 ^b^ ± 0.04
	Leaves	1.42 ^cd^ ± 0.02	1.62 ^a^ ± 0.08	1.49 ^bc^ ± 0.05	1.12 ^e^ ± 0.04	1.34 ^d^ ± 0.03	1.57 ^ab^ ± 0.04
Phenolics (mg g^−1^ DW)	Roots	6.70 ^a^ ± 0.78	4.40 ^b^ ± 0.69	4.50 ^b^ ± 0.70	1.40 ^d^ ± 0.36	2.0 ^cd^ ± 0.69	2.80 ^c^ ± 0.60
	Leaves	13.0 ^a^ ± 1.40	10.40 ^b^ ± 0.85	9.20 ^b^ ± 0.87	3.30 ^d^ ± 0.36	4.0 ^d^ ± 0.72	7.10 ^c^ ± 1.13

## Data Availability

The data presented in this study are available on request from the corresponding author.
